# Risk Factors and Outcomes of Heart Failure Following First-Episode of Acute Myocardial Infarction—A Case Series Study of 161,384 Cases

**DOI:** 10.3390/healthcare9101382

**Published:** 2021-10-16

**Authors:** Wen-Hwa Wang, Guang-Yuan Mar, Kai-Che Wei, Chin-Chang Cheng, Wei-Chun Huang

**Affiliations:** 1 Department of Cardiology, Kaohsiung Veterans General Hospital, Kaohsiung 813, Taiwan; whwang@vghks.gov.tw (W.-H.W.); gymar@vghks.gov.tw (G.-Y.M.); cccheng@vghks.gov.tw (C.-C.C.); 2 College of Management, I-Shou University, Kaohsiung 824, Taiwan; 3 Health Management Center, Kaohsiung Veterans General Hospital, Kaohsiung 813, Taiwan; 4 Shu-Zen Junior College of Medicine and Management, Kaohsiung 821, Taiwan; 5 Superintendent, Kaohsiung Municipal United Hospital, Kaohsiung 804, Taiwan; 6 Department of Dermatology, Kaohsiung Veterans General Hospital, Kaohsiung 813, Taiwan; kcwei@vghks.gov.tw; 7 School of Medicine, National Yang Ming Chiao Tung University, Taipei 112, Taiwan; 8 Department of Critical Care Medicine, Kaohsiung Veterans General Hospital, Kaohsiung 813, Taiwan; 9 Department of Physical Therapy, Fooyin University, Kaohsiung 831, Taiwan

**Keywords:** myocardial infarction, heart failure, National Health Insurance Research Database, retrospective cohort study, risk factors, survival outcome

## Abstract

Background: Heart failure (HF) is one of the important complications of acute myocardial infarction (AMI), but the epidemiology, associated risks and outcomes have not been well investigated in the era of broad use of fluoroscopy-guided angiographic intervention. Methods: We analysed 161,384 subjects who had experienced the first episode of AMI during 1 January 2000 and 31 December 2012 using the Taiwan National Health Insurance Research Database. Results: During the index AMI hospitalization, 23.6% of cases developed HF. Female, ≥65 years-old, non-ST-segment elevation type of MI, diabetes mellitus (DM), peripheral vascular occlusion disease (PAOD), chronic obstructive pulmonary disease (COPD), atrial fibrillation, and ventricular tachycardia/fibrillation (VT/VF) were associated with higher risks of developing HF. HF cases had inferior survival outcomes compared to non-HF cases in both the short and long term. Among those HF patients, ≥65 years, DM, PAOD, and VT/VF were associated with worse outcomes. On the contrary, coronary reperfusion intervention and treat-to-target pharmacologic treatment were associated with favourable survival outcomes. Conclusions: HF remains common in the modern age and poses negative impacts in survival of AMI patients. It highlights that prudent prevention and early treatment of HF during AMI hospitalization is an important medical issue.

## 1. Introduction

Heart failure (HF) is one of the most important complications of acute myocardial infarction (AMI). Despite the improvements of long-term survival after AMI, HF following AMI is occasionally noted in daily practice and associated with higher risk of mortality and higher readmission rate [1,2].

The incidence of HF following AMI has been broadly studied before but results were widely varied, ranging from 3.7 to 28% [3,4,5]. Aside from that, previous studies included a wide range of follow-up time after the index episode of AMI. It was difficult to conclude the exact percentage of HF directly caused by the index AMI because the aetiology of HF could be multifactorial. The longer the duration between AMI and the onset of HF is, the more difficult it is to prove that HF is mainly caused by AMI.

In addition, primary percutaneous coronary intervention (PCI) has been broadly used and became the main reperfusion intervention for ST-elevation myocardial infarction (STEMI). It is suggestive that early reperfusion for ischemic myocardium following AMI could reduce the likelihood of developing HF later [4,6]. Presumably, the incidences and prognosis of post-AMI HF may be different in the recent decade. However, the epidemiology, the associated risks and outcomes for post-AMI HF have not been well investigated in the modern era of PCI. Therefore, it is imperative to re-appraise the incidence and risk factors for prognosis of HF post AMI in the modern world.

This retrospective cohort study aimed to explore the epidemiology and prognosis of first ever HF complicating the first-time AMI using a nationwide health insurance database. We also analysed the impact of patients’ characteristics and treatment on the occurrence and survival outcomes of post-AMI HF.

## 2. Materials and Methods

This retrospective study complied with the guidelines of the Declaration of Helsinki and was approved by the Medical Ethics Committee of Veteran General Hospital, Kaohsiung, in Taiwan. The data was derived from the National Health Insurance Research Database (NHIRD) in Taiwan, which was launched on 1 March 1995. In Taiwan, more than 99.5% of the whole population of 23,000,000 people were enrolled in National Health Insurance with universal coverage. Institutional Review Board approval was obtained (IRB protocol number: VGHKS15-EM10-2) and waived the patient’s informed consent because all patient identification data were encrypted for privacy protection. All primary data were collected according to Strengthening the Reporting of Observational Studies in Epidemiology guidelines.

Adult cases (≥18 years-old) who had been admitted for the first ever episode of AMI (International Classification of Diseases, Ninth Revision, Clinical Modification [ICD-9-CM] codes 410–410.92) during 1 January 2000 and 31 December 2012 were included and were followed up until 31 December 2013. Those who had experienced previous history of HF (ICD-9-CM code 428) prior to the index admission were excluded. 

Patient characteristics were collected, including demographics, comorbidities (hypertension [IDC-9-CM codes 401–405], diabetes mellitus [ICD-9-CM code 250], dyslipidemia [IDC-9-CM code 272], peripheral vascular disease [ICD-9-CM codes 443.9, 441, 441.9, 785.4, V43.4, or procedure 38.48], chronic obstructive pulmonary disease (COPD) [ICD-9-CM code 491, 492, or 496], end-stage renal disease (ESRD) [ICD-9-CM code 585], stroke [ICD-9-CM codes 430–435, and 438], ventricular tachycardia (VT) [ICD-9-CM code 427.1], ventricular fibrillation (VF) [ICD-9-CM code 427.41]), and other associated clinical characteristics for AMI (types of AMI, coronary reperfusion therapy, and medications). The drug coding system was adopted from the WHO Anatomical Therapeutic Chemical Classification System (ATC code) to identify prescription medications during the admission of AMI. Survival was defined as the period starting from the date of admission of AMI to the end date of NHIRD coverage (31 December 2013).

### Statistical Analysis

Statistical data were obtained and analysed using SAS software version 9.4 (SAS Institute Inc., Cary, NC, USA). The Chi-squared test was employed to evaluate differences between categorical variables. The Kaplan–Meier estimator was used to compare different end points in AMI patients with HF and without HF, as well as to estimate the survival of those with HF or without HF in the long term. A multivariate Cox proportional hazard regression model was used to identify acute HF survival-associated factors after AMI onset by propensity score matching with demographics and comorbidity burden. *p*-value < 0.05 indicated statistical significance.

## 3. Results

**Figure 1:**
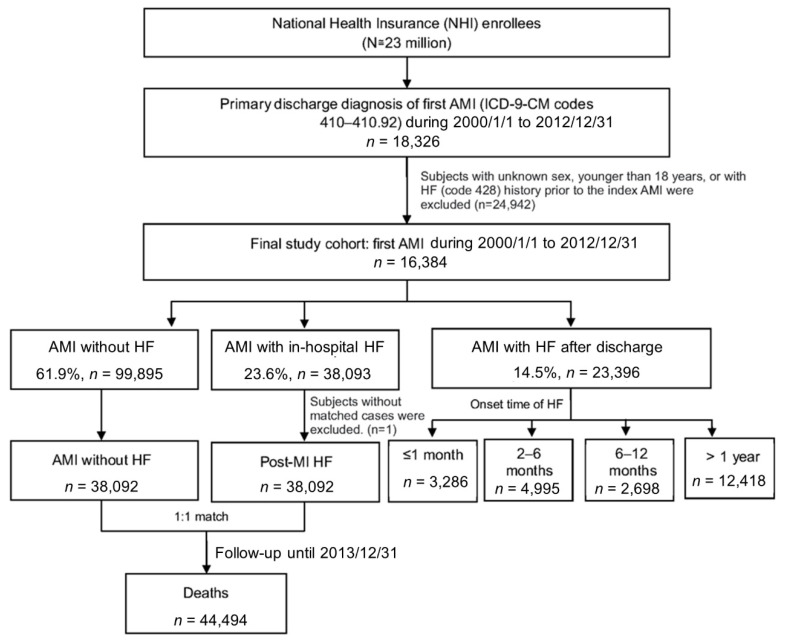
Study flow chart and the summary of the distribution of post-AMI cases in this study cohort.

A total of 161,384 cases had been admitted for their first-ever episode of AMI. Among them, 38,093 (23.6%) cases were diagnosed with HF during the index AMI admission (Figure 1). In addition, another 3286 (2.0%), 4995 (3.1%), 2698 (1.7%) and 12,418 (7.6%) cases had been admitted with the diagnosis of HF within 1 month, between 2–6 months, 6–12 months, and one year after discharge of AMI, respectively. (Figure 1)

The results revealed female gender (OR: 1.64, CI: 1.59–1.69), ≥65 years-old (OR: 2.32, CI: 2.26–2.38), NSTEMI (vs. STEMI, OR: 1.35, CI: 1.32–1.41), DM (OR: 1.54, CI: 1.50–1.57), peripheral vascular disease (OR: 1.65, CI: 1.55–1.76), ESRD (OR: 1.28, CI: 1.19–1.38), COPD (OR: 1.27, CI: 1.23–1.32), atrial fibrillation (OR: 1.65, CI: 1.58–1.73), and VT/VF (OR: 2.21, CI: 2.10–2.32) were significantly higher associated with development of post-MI HF during AMI admission (all *p* < 0.0001), whereas hypertension (OR: 0.96, CI: 0.93–0.98), dyslipidemia (OR: 0.72, CI: 0.70–0.73), and stroke (OR: 0.68, CI: 0.63–0.73) were negatively associated with development of HF (Table 1).

About the treatment and medication during AMI admission, a lower proportion of those who developed HF during AMI hospitalization received PCIs than these who did not experience HF (44.78 vs. 56.56%, *p* < 0.001). A very similar percentage of both groups received antiplatelets (such as aspirin, ticlopidine or clopidogrel) and ACEI or ARB. Notably, β-blockers (46.58 vs. 53.71%, *p* < 0.0001) and statins (31.77 vs. 38.99%) had been less prescribed, while spironolactone (22.23 vs. 5.76%) were more frequently prescribed for patients who developed HF during AMI admission (Table 1).

**Figure 2:**
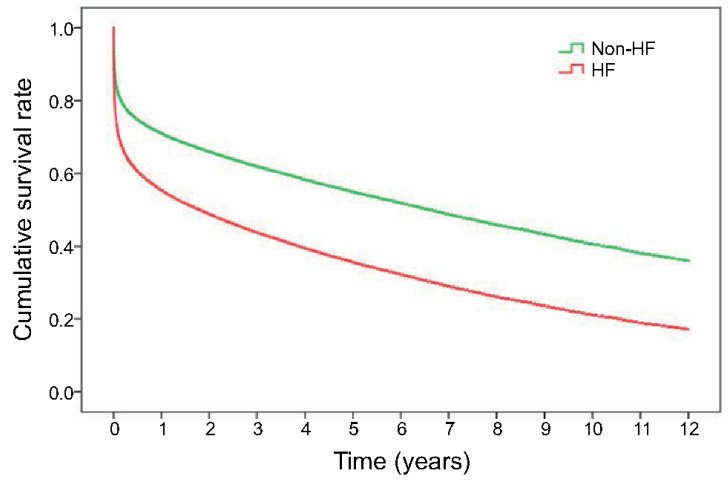
Kaplan–Meier curve presents long-term survival of the patients with or without HF after the first attack of AMI.

The Kaplan–Meier survival curves revealed both short-term and long-term survival rates were most favourable for the patients not experiencing HF. Patients diagnosed with HF at index admission had the worst survival rate (*p* < 0.0001) (Figure 2). In the multivariate Cox proportional hazard regression model, HF during index AMI hospitalisation was significantly associated with a higher risk of all-cause mortality (hazard ratio [HR] 1.85, 95% CI 1.81–1.88) after adjustment for demographics, intervention, comorbidities, and medications (Table 2). Medications prescribed during AMI hospitalisation may affect short- and long-term survival. Our study showed that patients receiving antiplatelet agents (HR 0.71, 95% CI 0.69–0.73), ACEI/ARB (HR 0.69, 95% CI 0.68–0.71), β-blockers (HR 0.83, 95% CI 0.81–0.85), and statins (HR 0.75, CI 0.73–0.85) had a better survival outcome.

Univariate analysis was used to investigate the risk factors for development of HF during the admission for AMI by performing 1:1 matching with those who developed HF and those who did not during AMI admission (Figure 1). 

**Figure 3:**
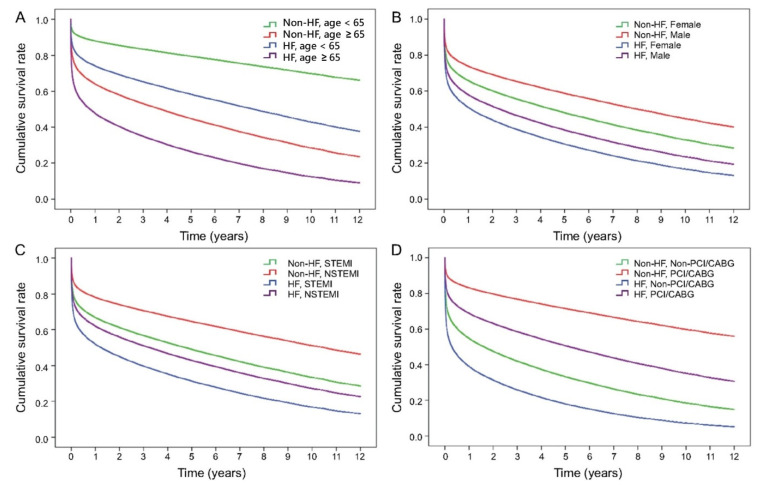
Subgroup analysis of survival curves for first ever AMI patients with or without HF in groups of different characteristics. (**A**) Age ≥ 65 and <65 years-old; (**B**) gender; (**C**) STEMI and NSTEMI; (**D**) receiving reperfusion intervention (PCI/CABG) or none.

We further analysed the impact of survival in different age (<65 or ≥65 years), sex, AMI types (STEMI or NSTEM), and receiving primary coronary reperfusion intervention (PCI or CABG). HF was invariably associated with a worse long-term survival rate in all subgroup analysis (Figure 3A–D). It is worth noted that HF patients who received PCI had a better survival than those without HF who did not receive PCI/CABG, while patients with HF who were not subjected to PCI/CABG showed the lowest survival rate (Figure 3D).

## 4. Discussion

To the best of our knowledge, this is one of the largest whole-population observational epidemiology studies that focused on first ever HF after the first episode of AMI in Asia. HF was common (23.6%) during hospitalization of the index AMI. Additionally, nearly one out of ten MI patients eventually developed HF after being discharged within 1 year. Female, older (age ≥ 65 years), and NSTEMI, diabetes mellitus, end-stage renal disease, COPD, and VT/VF were more vulnerable to develop HF. Notably, HF was a severe clinical manifestation associated with higher short- and long-term mortality in all groups of different sexes, ages, underlying comorbidities, and medications and cardiac resuscitation treatments. In the present study, diabetes mellitus, older age, hypertension, peripheral vascular disease, end-stage renal disease, stroke, COPD, and VT/VF increased the mortality rate, which is consistent with recent studies [7,8,9].

Our study reveals that post-MI HF is associated with a higher mortality rate in NSTEMI patients than in STEMI subjects (HR 0.94, 95% CI 0.92–0.96), which is in contrast to the findings of the study by Kaul et al. performed from 2002 to 2008, where the adjusted odd ratio of non-STEMI vs. STEMI for 1-year mortality was 1.0 (95% CI 0.9–1.2) [10]. We presume that the different results were due to the more widespread use of reperfusion interventions in two recent decades. Since the possibility of developing HF is associated with the infarct size [11], the reperfusion treatment brings benefits to the infarcted myocardium; as a result, reduction in infarct size and recovery of damaged myocardial tissues become more likely. Since primary PCI therapy has become the standard treatment for STEMI, the average HF severity and incidence has declined in STEMI patients who received primary PCI. However, NSTEMI patients tend to receive less reperfusion intervention compared to STEMI patients. That may explain why NSTEMI patients more frequently experience HF and have an inferior survival outcome.

It is debatable whether care quality and medications prescribed during the index hospitalisation of AMI can influence the development of HF. Our study revealed that the AMI patient groups with HF and without HF both had received a similar percentage of guideline-directed medication, including antiplatelet, and ACEI/ARB, which reflects that both HF and non-HF groups had received equal qualified standard care for AMI. On the contrary, fewer HF patients received β-blockers during the AMI admission than non-HF patients (46.58 vs. 53.71%). This might be attributable to the tendency of physicians to prescribe β-blockers less often for emerging acute HF. Nonetheless, HF patients received a higher percentage of spironolactone than non-HF patients (17.73 vs. 5.76%). This might be because spironolactone is one of the guideline-directed medications for HF patients but is not indicated for non-HF patients. Spironolactone improves survival in patients with HF. On the contrary, for AMI patients without HF, an early administration of spironolactone does not bring benefits of reducing adverse cardiac events in clinical trials [12,13].

Medications prescribed during AMI hospitalisation and coronary reperfusion intervention affect short-term and long-term survival. Antiplatelet agents (HR 0.71, 95% CI 0.69–0.73), ACEI/ARB (HR 0.69, 95% CI 0.68–0.71), β-blockers (HR 0.83, 95% CI 0.81–0.85), and statins (HR 0.75, CI 0.73–0.85) had a better survival rate. The adjusted HR for PCI/CABG was 0.48, which was lower than the adjusted HR for ACEI/ARB and β-blockers. It implies that AMI patients with HF obtain greater benefit from PCI/CABG than those who received drug treatment only and do not receive PCI/CABG during the index admission. Moreover, whether the patients had HF or not, those who received PCI/CABG had a better survival rate compared to those who did not. It is worth noting that HF patients who received PCI even had a better survival than those without HF who did not receive PCI/CABG (Figure 3D). In other words, AMI patients deserve to receive coronary reperfusion intervention during the index admission. However, myocardial ischemia-reperfusion injury caused by reperfusion therapy may lead to further cardiac tissue damage and paradoxical cardiomyocyte dysfunction. Since 1980s, intensive research in “reperfusion-induced injury” and its prevention was started and significantly increased under both experimental [14,15,16,17,18] and clinical conditions [19,20,21,22,23,24,25,26,27]. 

HF is a clinical syndrome with decreased cardiac output due to structural or functional cardiac disorders. Post-MI HF can be a consequence of myocardial necrosis, myocardial stunning, or complications such as papillary muscle rupture or ventricular rupture. Early detection of HF in AMI patients is important since HF may cause further multiorgan failure, ventricular tachycardia, or ventricular fibrillation [28]. Physicians should be alert to life-threatening ventricular arrhythmia for further management, including anti-arrhythmia medication or placing an implantable cardioverter-defibrillator in the future. Aside from that, post-AMI HF is associated with higher mortality rate, repeat admissions, lower quality of life, and higher economic burdens. It is debatable whether post-AMI HF is a reversible disorder or the start of a prolonged deterioration process. Fortunately, reperfusion therapy has played important roles in recovery from myocardial stunning, improvement of left ventricular systolic function, and better survival rate

Last, but not least, HF increases mortality after MI, irrespective of whether the onset is delayed or early [29]. HF following AMI can present as both early-onset and delayed-onset. The characteristics of the infarct, such as size, culprit lesion, and time-to-reperfusion, might be related to early-onset HF. Conversely, post AMI remodeling and subclinical ischemia are correlated with delayed-onset HF [30]. Physicians are encouraged be more aggressive in following up these patients, including prescribing and titrating medications, and adjusting treatment plans at different stages after discharge of AMI. Furthermore, a multidisciplinary team should care for and follow up with the patients as soon as possible.

### Study Limitations

A nationwide health insurance database cannot provide any detailed information on laboratory results, sites of coronary artery lesions, and size of infarcted areas in individual cases. Further, it was unlikely to distinguish the subtypes of HF, namely preserved ejection fraction HF and reduced ejection fraction HF.

## 5. Conclusions

New onset of HF following AMI remains common and is a predictor of a higher mortality. Female patients; older (≥65 years) patients; patients with diabetes mellitus, chronic obstructive pulmonary disease, or NSTEMI, and patients who have not undergone heart reperfusion intervention have a higher risk of HF development and long-term mortality. Physicians should be aware of HF signs and symptoms when treating AMI patients.

## Figures and Tables

**Table 1:** Basic characteristics of AMI patients associated with or without (in hospital) HF.

	No HF (*n* = 99,895)		HF (in Hospital) (*n* = 38,092)		OR	95% CI	*p* Value
Variables	*n*	(%)	*n*	(%)			
Sex							<0.0001
Male	74,491	(74.57)	24,097	(63.26)	1.00	—	
Female	25,404	(25.43)	13,486	(35.40)	1.64	(1.59–1.69)	
Age							<0.0001
<65	49,404	(49.46)	11,310	(29.69)	1.00	—	
≥65 years old	50,491	(50.54)	26,783	(70.31)	2.32	(2.26–2.38)	
Type							<0.0001
STEMI	43,486	(43.53)	13,830	(36.31)	1.00	—	
NSTEMI	56,409	(56.47)	24,263	(63.69)	1.35	(1.32–1.41)	
Comorbidities							
Hypertension	54,298	(54.36)	20,276	(53.23)	0.96	(0.93–0.98)	<0.0001
Dyslipidemia	48,079	(48.13)	15,193	(39.88)	0.72	(0.70–0.73)	<0.0001
Diabetes mellitus	32,322	(32.36)	16,131	(42.35)	1.54	(1.50–1.57)	<0.0001
Peripheral vascular disease	2552	(2.55)	1581	(4.15)	1.65	(1.55–1.76)	<0.0001
End-stage renal disease	2317	(2.32)	1125	(2.95)	1.28	(1.19–1.38)	<0.0001
Stroke	3192	(3.20)	832	(2.18)	0.68	(0.63–0.73)	<0.0001
COPD	9101	(9.11)	4311	(11.32)	1.27	(1.23–1.32)	<0.0001
Atrial fibrillation	5230	(5.24)	3190	(8.37)	1.65	(1.58–1.73)	<0.0001
VT/VF	3446	(3.45)	2782	(7.30)	2.21	(2.10–2.32)	<0.0001
Reperfusion intervention							
PCI	56,499	(56.56)	17,058	(44.78)	0.62	(0.61–0.64)	<0.0001
In-hospital medication							
Antiplatelets ^†^	85,264	(85.35)	32,918	(86.41)	1.09	(1.06–1.13)	<0.0001
ACEI or ARB	61,092	(61.16)	23,340	(61.27)	1.01	(0.98–1.03)	0.6954
Statin	38,949	(38.99)	12,104	(31.77)	0.73	(0.71–0.75)	<0.0001
Beta blocker	53,653	(53.71)	17,743	(46.58)	0.75	(0.73–0.77)	<0.0001
Spironolactone	5751	(5.76)	8469	(22.23)	4.68	(4.52–4.85)	<0.0001
Follow-up period years, mean (SD)	4.42	(3.80)	2.64	(3.19)	—	—	<0.0001

^†^ Antiplatelets included ticlopidine, aspirin, clopidogrel (CI, confidence interval; OR, odds ratio; COPD, chronic obstructive pulmonary disease; VT, ventricular tachycardia; VF, ventricular fibrillation; PCI, percutaneous coronary intervention; CABG, coronary artery bypass graft; ACEI, angiotensin-converting enzyme inhibitor; ARB, angiotensin II receptor blocker; SD, standard deviation).

**Table 2:** Association between basic characteristics and mortality for acute myocardial infarction patients using multivariate Cox proportional hazard regression analyses (n = 76,184).

Variables	Adjusted HR	95% CI	*p* Value
In-hospital HF (ref = no HF)	1.85	(1.81–1.88)	<0.0001
Male (ref = female)	1.04	(1.02–1.06)	<0.0001
age ≥ 65 years (ref = < 65 years)	2.35	(2.30–2.42)	<0.0001
STEMI (ref = NSTEMI)	0.94	(0.92–0.96)	<0.0001
Comorbidities			
Hypertension	1.07	(1.05–1.09)	<0.0001
Dyslipidemia	0.94	(0.91–0.97)	<0.0001
Diabetes mellitus	1.28	(1.26–1.31)	<0.0001
Peripheral vascular disease	1.46	(1.41–1.53)	<0.0001
End-stage renal disease	1.52	(1.45–1.60)	<0.0001
Stroke	1.19	(1.12–1.26)	<0.0001
COPD	1.16	(1.13–1.19)	<0.0001
Atrial fibrillation	0.97	(0.94–1.00)	0.0381
VT/VF	1.33	(1.28–1.38)	<0.0001
Primary reperfusion intervention			
PCI/CABG	0.48	(0.47–0.49)	<0.0001
In-hospital medication			
Antiplatelets ^†^	0.71	(0.69–0.73)	<0.0001
ACEI or ARB	0.69	(0.68–0.71)	<0.0001
Statin	0.75	(0.73–0.85)	<0.0001
Beta blocker	0.83	(0.81–0.85)	<0.0001
Spironolactone	1.04	(1.01–1.06)	0.0114

^†^ Antiplatelets included ticlopidine, aspirin and clopidogrel (CI, confidence interval; OR, odds ratio; VT, ventricular tachycardia; VF, ventricular fibrillation; PCI, percutaneous coronary intervention; CABG, coronary artery bypass graft; ACEI, angiotensin-converting enzyme inhibitor; ARB, angiotensin II receptor blocker).

## Data Availability

The data that support the findings of this study are available from the National Health Insurance Research Database (NHIRD) but restrictions apply to the availability of these data, which were used under license for the current study, and so are not publicly available.

## List of Abbreviations

ACEI/ARB	Angiotensin-converting enzyme inhibitors/angiotensin II receptor blockers
AMI	acute myocardial infarction
CABG	coronary artery bypass graft
HF	heart failure
NHIRD	National Health Insurance Research Database
PCI	percutaneous coronary intervention
NSTEMI	Non-ST elevation myocardial infarction
STEMI	ST elevation myocardial infarction
VT/VF	ventricular tachycardia/ventricular flutter
